# All-cause mortality in the Aberdeen 1921 birth cohort: Effects of socio-demographic, physical and cognitive factors

**DOI:** 10.1186/1471-2458-8-307

**Published:** 2008-09-10

**Authors:** John M Starr, Ian J Deary, Lawrence J Whalley

**Affiliations:** 1 Geriatric Medicine Unit, University of Edinburgh, Edinburgh, UK; 2 Psychology, University of Edinburgh, Edinburgh, UK; 3 Department of Environmental and Occupational Medicine, University of Aberdeen, Foresterhill, Aberdeen AB25 2ZD, UK

## Abstract

**Background:**

Childhood intelligence predicts mortality throughout most of the life span. However, it is unknown whether its effect persists into advanced old age.

**Methods:**

The Aberdeen Birth Cohort born in 1921 (n = 354) and that had an IQ test as part of the national Scottish Mental Survey of 1932 were seen in 1997 at age 76 years when childhood and adult socio-environmental, medical and cognitive data were collected. Participants were followed until May 2007 and vital status determined from the General Register for Scotland records. Univariate associations between baseline variables and mortality were determined and multivariable survival analysis performed with Cox's proportional hazards modelling.

**Results:**

One hundred and fifty-eight (44.6%) of the 354 cohort members had died by the census date. Significantly more men (n = 102) died during follow-up than women (n = 56, χ^2 ^= 5.27, p = .022). Lower scores on four of the six cognitive tests at age 76 years were associated with increased mortality, but not IQ age 11. Survival was associated with gender (H.R. 0.32, 95% C.I. 0.11–0.89 for women versus men), peak expiratory flow rate (H.R. 0.997, 95% C.I. 0.992–1.001 per l/min) and the Uses of Common Objects test (H.R. 0.91, 95% C.I. 0.82–1.01)

**Conclusion:**

Both physical and psychological variables independently predicted survival in old age: respiratory function and executive function in particular. Male gender conferred increased risk of mortality and this was not explained by the broad range of socio-environmental, mental ability and health status variables examined in the study.

## Background

A wide range of health, lifestyle and cognitive variables influence mortality [1]. Factors in old age that impact on survival are frequently related to life-long tendencies. Childhood intelligence would be an example of this with regard to cognitive variables in adulthood [2], though its effects may lessen for cardiovascular causes of mortality over the age of 65 [3]. In addition, there is a distorting factor that affects mortality of older Scottish men between 1980 and 2002: World War II. Paradoxically, men in the highest quartile for childhood IQ were significantly more likely to die during the war [2]. This means that there are relatively fewer men from this upper IQ quartile available to survive beyond 75, so that within the population the overall protective effect of higher IQ on survival would be expected to be less.

In Scotland cardiovascular disease is the greatest contributor to all-cause mortality [4]. Reductions in cardiovascular mortality are attributable both to modern treatments (medical and surgical) and to improvements in population risk factors (blood pressure, cholesterol and smoking) [4]. The relatively smaller improvements in mortality in those aged over 75 years may reflect a lack of evidence-based practice in this age group that has been historically poorly represented in heart disease trials [5]. Who receives such survival-improving treatment may reflect a combination of current behaviours (e.g. smoking) and life-long traits (e.g. education and social class). This may partly explain how socio-economic disadvantages persist into old age [6]. Moreover, effects of socio-economic disadvantage may be amplified by new population behaviours. For example, the increased prevalence of smoking among women that coincides with the epochs of recent mortality studies, has had a major effect [7]. On the other hand, discrete exposures may have age-dependent effects. An example is that non-cancer mortality related to the atomic bombings of Hiroshima and Nagasaki was highest in those aged 30–49 years in 1945 [8]. Since most people die in old age, determining the risk factors for mortality in older people is important, but requires a life-course approach to identify key influences on survival.

## Methods

### Sample

ABC1921 participants were all born in 1921 and underwent an IQ-type test (the Moray House Test No 12) in a Scottish-wide survey of schoolchildren in June 1932. Full details of the ABC1921 study design and test battery are published elsewhere [2.9,10,11] but in 1997, with permission from Grampian Research Ethics Committee, at the start of "Wave 1" of the ABC1921 study, 297 individuals living in or near Aberdeen were identified and 234 agreed to be assessed. By Wave 5, which was conducted between March 2003 and March 2004, 107 individuals were still in touch with the study. The median dates (participant numbers) for the first, second, third, fourth and fifth Waves respectively were May 1998 (234), September 1999 (207), March 2001 (162), March 2002 (146) and August 2003 (107). It should however be noted that 47 individuals were recruited to the ABC1921 cohort for the first time in Wave 2 and a further 16 were recruited in Wave 3 as refreshment of the sample in early Waves.

### Measures

#### Mental ability

The 1932 Scottish Mental Survey [12] provides unique pre-morbid mental ability data relevant to a cohort at risk of age-related diseases. Under the auspices of the Scottish Council for Research in Education (SCRE), all children at school in Scotland on June 1^st ^1932 and born in the calendar year 1921 undertook a group-administered mental ability test, including some practice items. 87,498 (44,210 boys and 43,288 girls) were tested on this Moray House Test (MHT) that comprised a wide range of items with a maximum score of 76 and includes verbal reasoning, numerical, spatial and other items [10]. The scores on the 1932 Moray House Test were validated by individually re-testing a representative sample of 1000 of the children (500 boys, 500 girls) on the Stanford Revision of the Binet-Simon test. In addition, at first attendance as adults the following cognitive tests were administered: Mini-Mental State Examination (MMSE – a general cognitive screening test), Raven's Progressive Matrices (RPM – a measure of non-verbal reasoning), Rey Auditory Verbal Learning Test (AVLT – a measure of verbal declarative memory), Wechsler Adult Intelligence Scale subtests Block Design (BD – a measure of visuo-spatial ability) and Digit Symbol coding (DS – a measure of processing speed), and Uses of Common Objects Test (UCO – a measure of executive function) as detailed previously [11].

#### Demographic

We recorded data on marital status, living group, postal address, usual occupation (before retirement) and years of education. Occupations were classified using the UK Registrar General's Classification of Occupations [13] (1990). We used the ecological method of Carstairs and Morris [14] (1990) to assign a socio-economic deprivation index to each postal address. This method has advantages over occupational classification especially among older women. The usual paternal occupation (or breadwinner) in 1932 was recorded and classified as either (1) professional administrative; (2) skilled manual or (3) unskilled manual or unemployed [15].

#### Environmental

The number of persons living in the home at age 11, the number of public rooms in the home, whether at age 11 the participant was routinely required to share a bed (BS), indoor or outdoor sanitation (SN) and how many people shared this toilet were recorded (SS). An "*overcrowding index*" (OI) was derived by dividing the total number of usual residents in the house by the number of public rooms.

#### Health status

Information on disease history and prescribed medication was recorded at interview. The research nurse completed a clinical examination that included pulse, systolic and diastolic BP (mean of three occasions, sitting), height, weight, best of three of Forced Expiratory Volume in one second (FEV_1_), Forced Vital Capacity (FVC) and Peak Expiratory Flow Rate (PEFR) measured using a microspirometer. Subjects were classified as never smoker, past smoker, or current smoker. In addition we administered the Hospital Anxiety and Depression scales (HADS) scoring on both anxiety and depression subscales [16].

#### Vital status

This was determined for a census date of 12 May 2007 from Scottish Community Health Indices (CHIs). Deaths are flagged on each CHI from the General Register Office for Scotland via computerised links provided by ISD Scotland. However the vital status of participants no longer listed on Scottish CHIs remained undetermined.

### Statistical analysis

After initial data checking and description, univariate associations with mortality were explored using Chi-square test (with continuity correction where appropriate) or Fisher's exact test for categorical variables and analysis of variance or Wilcoxon's test for non-categorical variables. Following this Cox's proportional hazards modelling was performed. As a first step the effect of age was tested for in this narrow-age cohort. Then models were built according to the proportion of participants with available data for each variable, entering those variables with least missing data first. This resulted in socio-demographic variables being examined prior to cognitive variables. At each stage we applied backward elimination with p > .1 criterion for variable removal. All analyses were undertaken using the SPSS 14.0 statistical package.

## Results

### Description of sample

The cohort comprised 354 (202 male, 152 female) members all born in 1921. Table 1 shows baseline characteristics. One hundred and fifty-eight (44.6%) of the 354 cohort members had died by the census date. Seventeen members moved from the area during follow up, the vital status of five of these was unknown at the census date and excluded from analyses.

**Table 1:** Baseline characteristics of the ABC1921 participants; cognitive and physical measures are reported for those attending at Wave 1.

Variables	N	Mean	Standard deviation
Demographic and environmental			
**Education (years)**	296	9.8	1.8
**Number of rooms in family home age 11**	209	3.4	1.8
**Number of people resident in family home age 11**	212	5.6	1.8
**Number of people sharing sanitation facilities age 11**	209	9.9	6.4
**Deprivation score at first attendance**	348	3.2	1.4
Health status			
**Units of alcohol consumed per week at first attendance**	290	5.3 (median 1.0)	9.2 (interquartile range 7.0)
**Height (cm)**	185	162.1	8.7
**Weight (kg)**	189	67.9	12.6
**Demi-span**	199	83.9	5.1
**Systolic BP (mmHg)**	206	164	26
**Diastolic BP (mmHg)**	206	88	15
**PEFR (l/min)**	193	297	111
**FEV**_1 _**(l)**	192	1.85	.59
**FVC (l)**	193	2.13	.71
**Visual acuity of best eye**	182	6/9	N/A
**6 m walk time (s)**	182	6.8	2.1
**HADS anxiety score**	221	5.1	3.1
**HADS depression score**	221	3.7	2.9
**Barthel score**	234	19.4	1.6
Mental ability			
**MHT age 11**	327	37.7	13.5
**MMSE**	236	28.1	2.2
**RPM**	200	27.1	9.0
**AVLT**	91	50.2	14.4
**BD**	89	19.5	7.7
**DS**	92	31.0	10.8
**UCO**	82	16.8	7.7

### Univariate associations with mortality

Significantly more men (n = 102) died during follow-up than women (n = 56, χ^2 ^= 5.27, p = .022). Table 2 shows sex-adjusted univariate associations for the other demographic and environmental variables with mortality: none were significant. Table 3 shows sex-adjusted univariate associations for the mental ability variables with mortality: lower scores on MMSE, RPM, AVLT and DS were associated with increased mortality risk. MHT age 11 was not significantly associated with mortality in this cohort aged 76 years at baseline. Table 4 shows sex-adjusted univariate associations for the health status variables with mortality: increased mortality risk was significantly associated with a history of heart disease, lighter weight, poorer respiratory function, being a current smoker, higher anxiety and higher depression scores.

**Table 2:** Sex-adjusted univariate associations of demographic and environmental variables with mortality.

Variable	Odds ratio	95% confidence intervals
**Age at wave 1**	0.56 per year	0.27–1.15
**Education**	0.87 per year	0.75–1.01
**Occupation**	1.08 per group	0.98–1.20
**Deprivation**	1.07 per category	0.92–1.26
**Living alone**	0.81 alone versus cohabiting	0.49–1.35
**Paternal occupation**	1.03 per class	0.78–1.34
**Number of rooms in house age 11**	0.90 per room	0.75–1.07
**Number of people resident in house age 11**	1.06 per person	0.90–1.23
**Crowding index age 11**	1.17	0.88–1.59
**Sanitation age 11**	0.77 indoors versus outdoors	0.41–1.44
**Number of people sharing sanitation age 11**	1.03 per person	0.98–1.08
**Bed sharing age 11**	0.81 sharing versus non-sharing	0.43–1.54

**Table 3:** Sex-adjusted univariate associations of Wave 1 mental ability variables with mortality.

Variable	Odds ratio	95% confidence intervals
**MHT age 11**	0.99 per point	0.98–1.01
**MMSE**	0.68 per point	0.57–.082
**RPM**	0.95 per point	0.92–0.98
**AVLT**	0.96 per point	0.93–0.99
**BD**	0.94 per point	0.88–1.002
**DS**	0.96 per point	0.92–0.998
**UCO**	0.92 per point	0.84–1.01

**Table 4:** Sex-adjusted univariate associations of Wave 1 health status variables with mortality.

Variable	Odds ratio	95% confidence intervals
**History of any disease**	1.07	0.23–4.94
**Any medicine prescribed**	1.01	0.56–1.83
**History of heart disease**	1.80	1.07–3.03
**History of hypertension**	0.66	0.40–1.11
**History of diabetes**	1.70	0.61–4.74
**Height**	0.99 per cm	0.94–1.04
**Weight**	0.97 per kg	0.94–0.996
**Systolic BP**	1.0 per mmHg	0.99–1.01
**Diastolic BP**	0.99 per mmHg	0.97–1.01
**PEFR**	0.996 per l/min	0.993–0.999
**FEV**_1_	0.42 per l	0.23–0.76
**FVC**	0.76 per l	0.47–1.22
**Smoking habit**	0.96 ex-smoker	0.56–1.63
	6.12 current smoker	2.27–16.5
**Alcohol use**	1.0 per unit	0.97–1.03
**HADS anxiety score**	1.12 per point	1.02–1.22
**HADS depression score**	1.18 per point	1.06–1.31

### Cox's proportional hazards models

In a univariate model, women were significantly less likely to die (Hazards ratio [H.R.] 0.67, 95% confidence intervals [C.I.] 0.48–0.94). Once sex was adjusted for, age at Wave 1 was not significantly associated with survival (n = 353, p = .60) in this narrow-age cohort. Next a backward elimination model for demographic variables (education, occupation, deprivation, marital status and living alone, n = 288 with complete data) was examined: gender was identified as the only significant predictor of mortality. Similarly a backward elimination model for childhood variables (paternal social class, number of rooms, number of residents, crowding index, sanitation location, number sharing sanitation facilities, sharing a bed, n = 197 with complete data) identified gender as the only significant predictor of survival. A similar model for the mental ability variables identified UCO as a significant predictor (n = 80, H.R. 0.90, 95% C.I. 0.84–0.98) in addition to gender. For the model including health status variables (n = 158 with complete data), in addition to gender, significant predictors were current smoker (H.R. 2.19, 95% C.I. 1.06–4.53), HADS depression score (H.R. 1.08, 95% C.I. 1.00–1.16) and PEFR (H.R. 0.997, 95% C.I. 0.995–1.00). In a combined backward elimination model retaining those variables identified as significant above (gender, UCO, smoking, depression score and PEFR, n = 63) only gender (H.R. 0.32, 95% C.I. 0.11–0.89 for women versus men), PEFR (H.R. 0.997, 95% C.I. 0.992–1.001) and UCO (H.R. 0.91, 95% C.I. 0.82–1.01) were in the optimal model as judged by overall χ^2^, though PEFR and UCO were not significant, only showing a statistical trend.

## Discussion

In this cohort of Scots born in 1921 survival beyond 76 years related to gender, cognition and physical health measures. The very narrow age range meant that chronological age, itself, did not predict survival. Furthermore, early-life influences, such as childhood IQ, did not have any significant effect in contrast to younger cohorts [2,3]. Neither did variables relevant to earlier adult life such as education and occupation. Lower scores on several of the Wave 1 mental ability variables were associated with poorer survival: in a multivariate model UCO, a measure of executive function, was the best cognitive predictor. Amongst health variables, multivariate models showed that conventional measures of health status, such as diagnosed disease and medication use, were poor predictors of survival. Key health status predictors were respiratory function, mood score and smoking habit. The survival of participants who had given up smoking was no worse than those who had never smoked. Those who continued to smoke were more than twice as likely to die during the nine years follow-up. Lower mood was an important predictor of mortality even within the 'normal' range. A combined model showed that the major predictor of mortality at this age remained gender, even after other socio-demographic, cognitive and health status variables were adjusted for.

A study of 114 people aged 70 years and older from a Dutch population found that dyspnoea scores were significant predictors of death over an eight year follow-up period [17]. A systematic review of the associations between pulmonary function and cardiovascular mortality comprising a combined sample of 83,880 adults of all ages found that those in the lowest quintile of FEV_1 _had nearly double the risk of dying compared with those in the highest quintile [18]. Our data are consistent with these findings and, in addition, show that respiratory function is a significant predictor independent of smoking habit, socio-environmental or early life influences. Smoking was related to survival in older men in the Western Collaborative Study [19], though this was unadjusted for pulmonary function. Smoking was a significant predictor of mortality independent of lung disease in the Asset and Health Dynamics Among the Oldest Old Survey [20]. Neither of these studies differentiated between current and ex-smokers. Our data suggest that smoking beyond 76 years of age continues to incur an increased risk of dying. Impaired cognition is also well recognised as a predictor of death, even when a wide range of other covariables are adjusted for [21]. Within the range of mental abilities, non-verbal tests were better predictors of survival in 546 non-institutionalised Texans aged over 70 years, in particular an executive clock-drawing task [22]. We also found a range of mental abilities associated with increased mortality, with executive function most strongly associated in multivariable models. It is possible that these findings reflect the influence of a key underpinning cognitive function, such as represented by reaction time that, itself, predicts survival [23].

The study is limited in several ways. First, although the narrow age range ensures that exposures are very similar for all participants (e.g. occupation, education, childhood experiences), it makes generalisation more difficult. For example, the majority of participants had outdoor sanitation at age 11, a situation very different in Aberdeen today. Secondly, the sample size means that important effects in small sub-groups could be missed. This is particularly relevant for multivariate models that included UCO where power was substantially reduced and consequently type 2 statistical error more likely. For this reason we did not investigate potential interactions (e.g. between sex and smoking). Thirdly, some variables that might be important in other populations were not considered (e.g. ethnic group – the sample is uniformly Caucasian). Fourthly, we chose backward elimination based on likelihood ratio change. Alternative strategies may have identified different predictors. Moreover, we did not include a time-dependent term in the models for much the same reason as that for interactions not being considered. Fifthly, the vital status for just over 1% of the participants was unknown at the census date because they had moved, though we had no evidence for their deaths being registered in Scotland. It is possible that they had emigrated from Scotland and died elsewhere. However, even if all five had died, it is unlikely to have altered the results substantially.

One advantage of a narrow age cohort is that key predictors of mortality can also be considered as measures of biological age. That is, although all the participants are almost identical in chronological age, some are at greater risk of dying than others. These individual differences in survival can be used as one criterion for biological age. In this cohort conventional health status items, such as diagnoses, were not good measures of biological age by this criterion. Respiratory function and cognition were better measures. But even after accounting for physical and mental health status, women were significantly biologically younger than men. Further investigations are required to elucidate the mechanisms underpinning this observation.

## Conclusion

Mortality was common beyond 76 years of age in the ABC1921 cohort. Many socio-environmental variables associated with premature mortality no longer predicted death at this age. However, continuing to smoke still more than doubled the risk of dying. Both physical and psychological variables independently predicted survival in old age: respiratory function and executive function in particular. Male gender conferred increased risk of mortality and this was not explained by the broad range of socio-environmental, mental ability and health status variables examined in the study.

## Competing interests

The authors declare that they have no competing interests.

## Authors' contributions

All authors contributed equally to the design, analysis and reporting of the study.

## Pre-publication history

The pre-publication history for this paper can be accessed here:


